# Supplementary material for: Dynamic changes in carbonate chemistry in the microenvironment around single marine phytoplankton cells
Source: Nat Commun. 2018 Jan 8;9:74. doi: 10.1038/s41467-017-02426-y (PMC5758611; doi:10.1038/s41467-017-02426-y)
Supplement: Supplementary file 1 — Supplementary Information [file 41467_2017_2426_MOESM1_ESM.pdf]

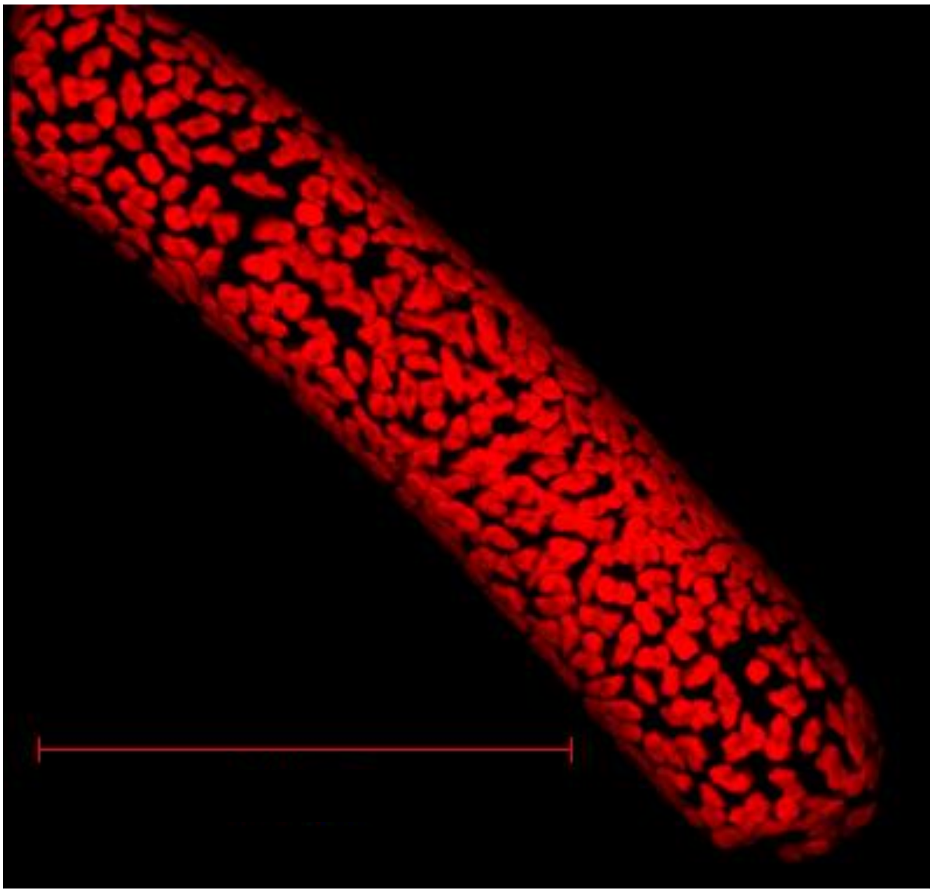

Supplementary Figure 1: Distribution of chloroplasts in *O. sinensis*

Confocal microscopy of an *O. sinensis* cell indicates that chloroplasts are evenly distributed along the length of the cell. A 3D projection of chlorophyll fluorescence is shown, generated from a Z-stack of 30 images (1 µm per slice). Scale bar represents 100 µm.

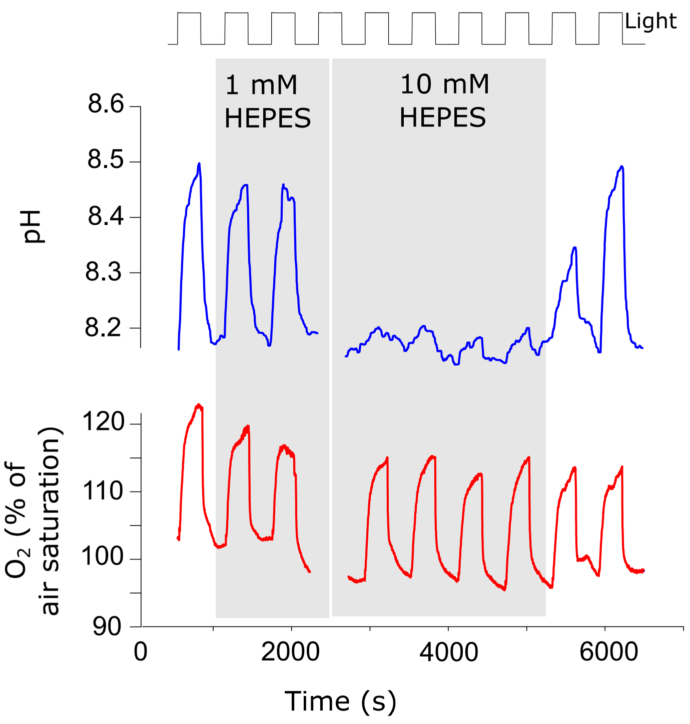

Supplementary Figure 2: The effects of buffers on cell surface pH

Light-dependent changes in cell surface pH and [O_2] were measured around an *O. sinensis* cell in normal unbuffered ASW media, ASW + 1 mM HEPES and ASW + 10 mM HEPES (all at pH 8.2). The addition of 10 mM HEPES almost completely buffers the light-dependent increases in cell surface pH, although there is little impact in photosynthetic O_2 evolution. The trace shown is representative of 10 individual cells examined.

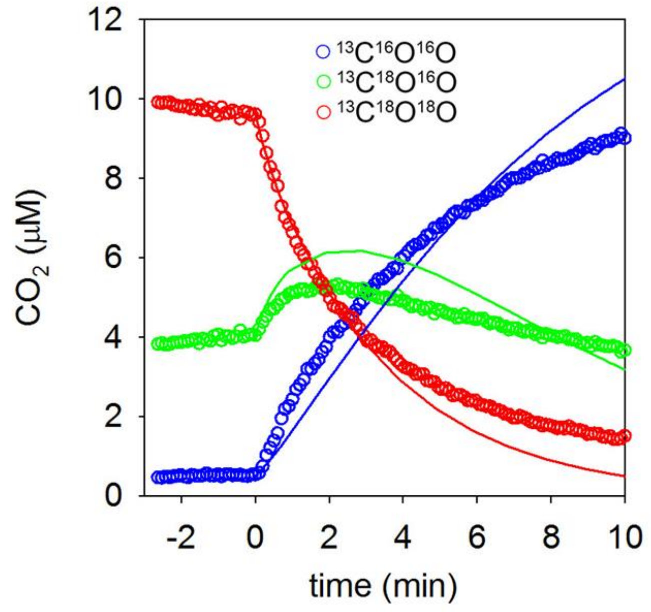

Supplementary Figure 3: Estimation of eCA activity in *O. sinensis* using MIMS

The removal of ^{18}O from labelled CO_2 and HCO_3^- is accelerated upon the addition of *O. sinensis* cells at t=0. The lines show a model fit to the data used to infer absolute eCA activity from ^{18}O removal dynamics.

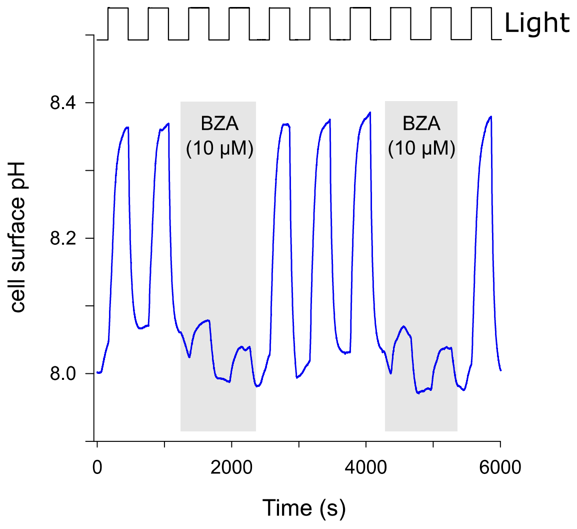

Supplementary Figure 4: Inhibition of eCA by benzolamide.

Rapid inhibition of the light-dependent increase in cell surface pH in *O. sinensis* by 10 µM benzolamide (BZA). pH was monitored at the surface of a single *O. sinensis* cell. The effect of 10 µM BZA was reversible, with all cells exhibiting a rapid recovery from inhibition. The trace shown is representative of 8 cells examined.

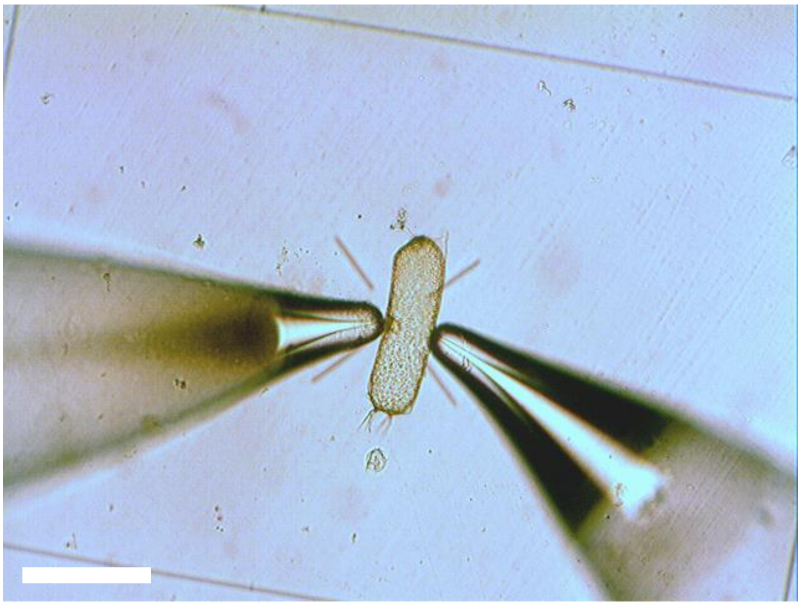

Supplementary Figure 5: Positioning of microelectrodes around an *O. sinensis* cell for simultaneous measurement of pH and CO_3^{2-}

Custom-made microelectrodes were positioned using micromanipulators against the frustule in the central region of an *O. sinensis* cell. For simultaneous measurement of pH and [CO_3^{2-}], the respective ion-selective microelectrodes were positioned on opposing sides of the cell, which had the added benefit of stabilising the cell. The image shows blunt-ended microelectrodes that were used with the larger diatom species (*O. sinensis* and *Coscinodiscus*). Blunt-ended electrodes were produced by fire-polishing and have an approximate external tip diameter of 20 µm. The internal tip diameter of the sensor is much smaller and is around 1-2 µm. Scale bar = 100 µm.

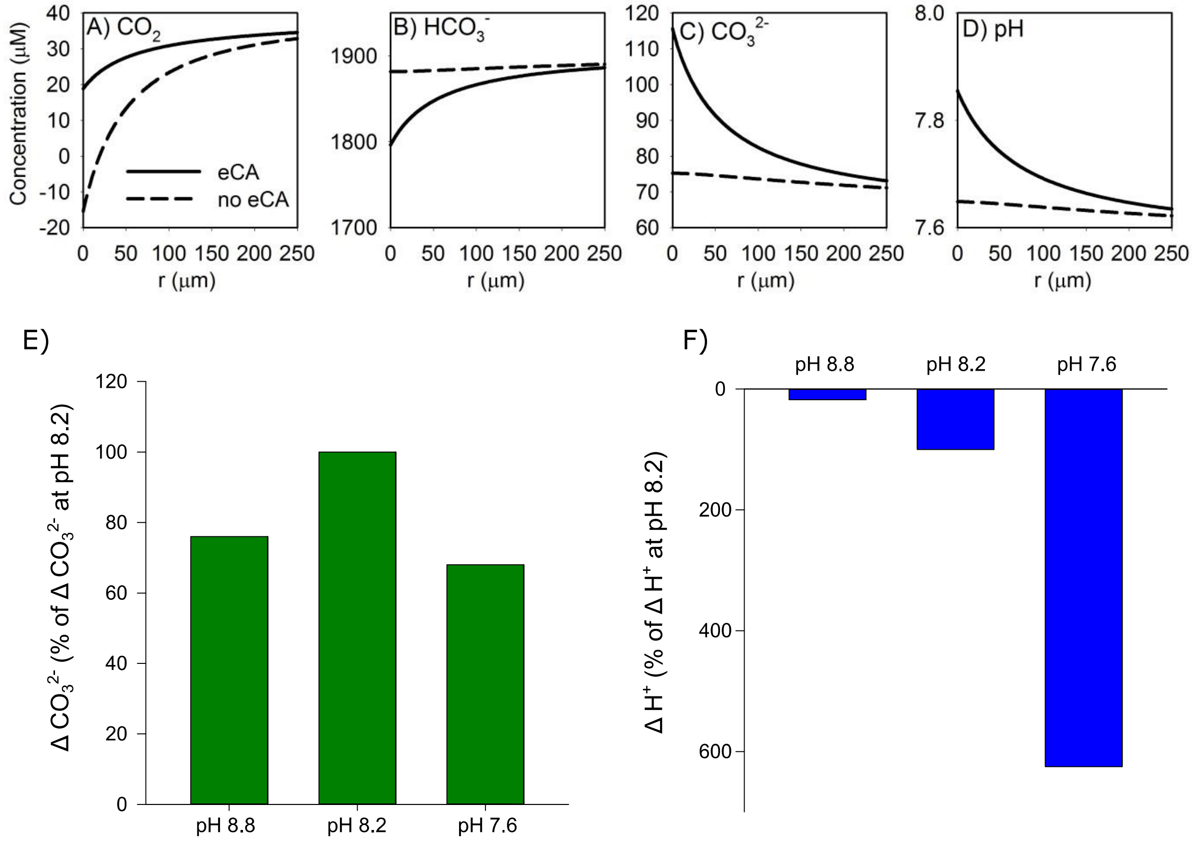

Supplementary Figure 6: Cellular modelling of carbonate chemistry around a large diatom cell at high CO_2.

A-D) Modelled profiles of inorganic carbon species (CO_2, HCO_3^-, CO_3^{2-}) and pH around a large (r = 60 µm) photosynthesizing cell at high CO_2 (pH = 7.6). The model simulates a cell taking up only CO_2 for photosynthesis. The horizontal axis represents distance away from the cell surface. The results show that even at high CO_2 concentrations eCA activity is required to support the simulated rates of CO_2 uptake. E) Simulated changes in cell surface [CO_3^{2-}] for a cell at bulk seawater pH of 7.6, 8.2 or 8.8 (based on the experiment described in Fig. 6). F) Simulated changes in cell surface [H^+] for a cell at bulk seawater pH of 7.6, 8.2 or 8.8.

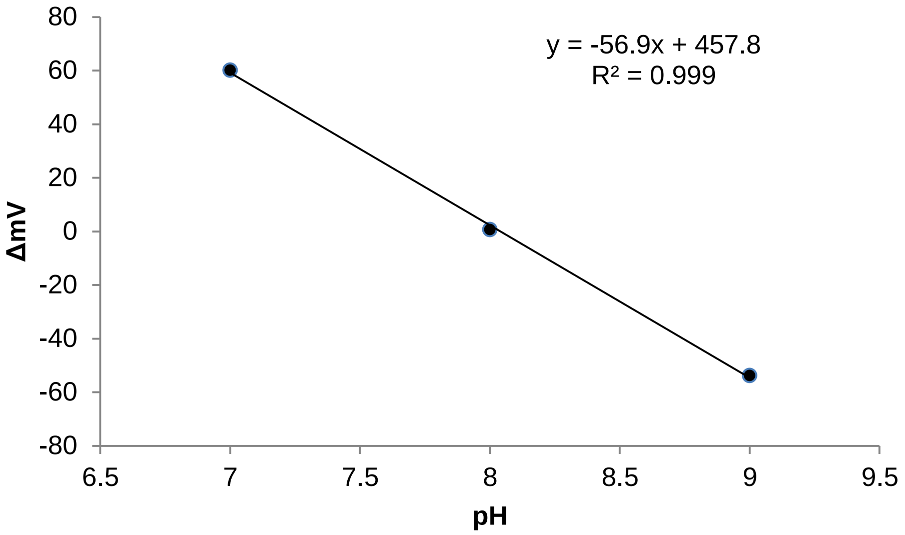

Supplementary Figure 7: Calibration of pH electrodes

The pH microelectrodes were calibrated using a 3-point calibration using seawater solutions at pH_{NBS} 7.0, 8.0 and 9.0. The microelectrode in the example show exhibited a linear response to changes in pH with a slope of -56.9 mV/pH unit.

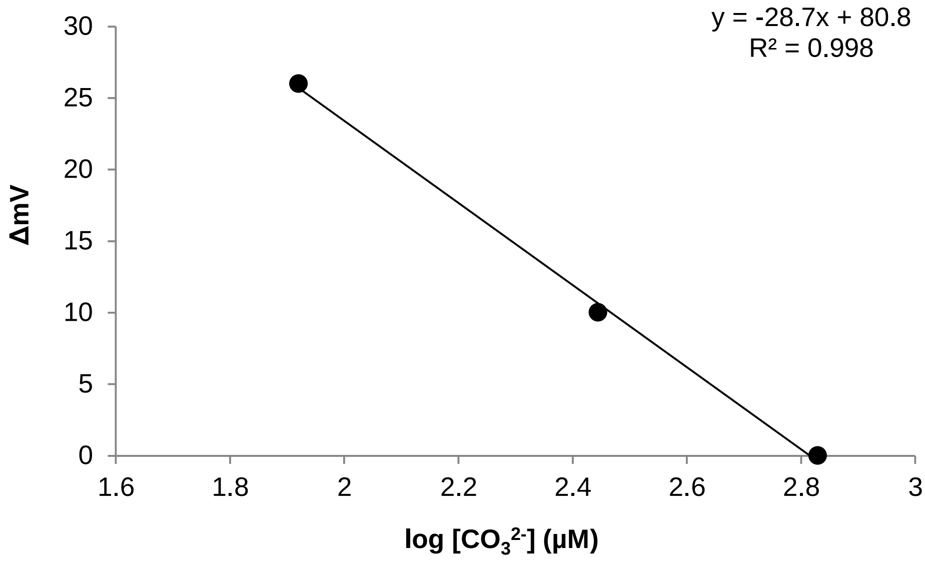

Supplementary Figure 8: Calibration of CO_3^{2-} microelectrodes

The CO_3^{2-} microelectrodes were calibrated using a 3-point calibration. Seawater solutions were adjusted sequentially to pH_{NBS} 8.8, 8.2 and 7.6 by bubbling with CO_2. Seawater pH was monitored continuously during this process using a commercial Ross glass electrode. Samples were taken for total alkalinity (TA) analysis after the pH had stabilised. CO_3^{2-} was calculated from pH and TA using CO2SYS. The microelectrode in the example shown exhibited a log-linear response over the range of [CO_3^{2-}] examined, with a slope of -28.7 mV/decade.

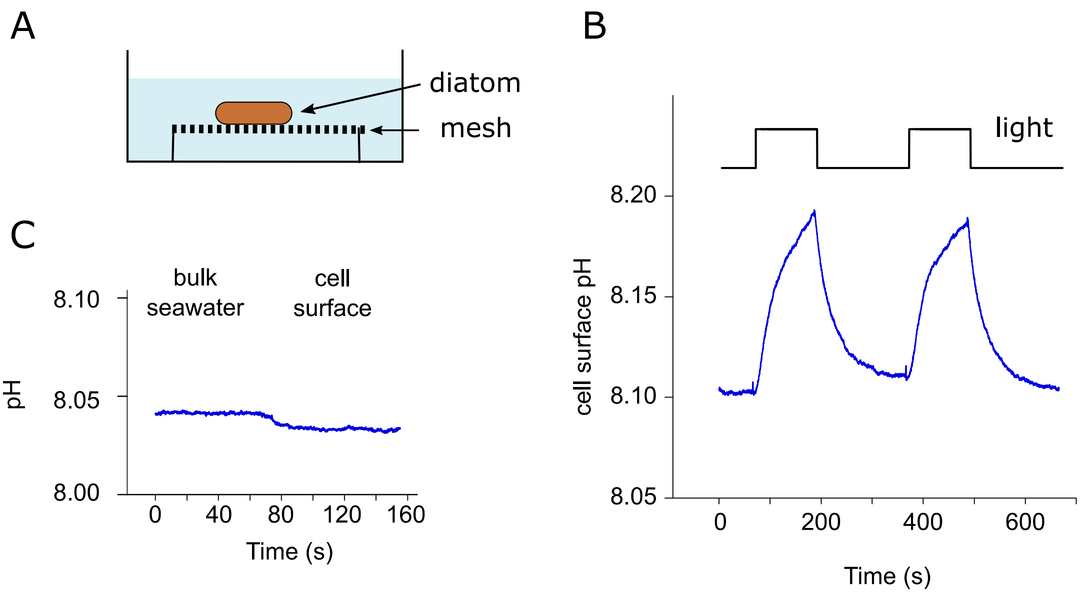

Supplementary Figure 9: Support of cells on a fine mesh

To test whether placing cells on directly onto a glass-bottomed dish significantly influenced the formation of the diffusion boundary layer around cells, cell surface pH was also measured around cells that were suspended on a fine mesh (pore size 100 µm). A) Schematic view of the experimental setup. B) Light-dependent changes in cell surface pH around a single *O. sinensis* cell. Irradiance 100 µmol m^{-2} s^{-1}. The trace shown is representative of four cells examined. C) Measurement of cell surface pH using a suspended *O. sinensis* cell in the dark compared to bulk seawater. There is a very small decrease in cell surface pH as the microelectrode is positioned against the cell surface. Metabolic processes in the dark therefore only have a minor influence on cell surface pH. A representative trace is shown from three cells examined.
